# A radical revision of the public health response to environmental crisis in a warming world: contributions of Indigenous knowledges and Indigenous feminist perspectives

**DOI:** 10.17269/s41997-020-00388-1

**Published:** 2020-08-06

**Authors:** Diana Lewis, Lewis Williams, Rhys Jones

**Affiliations:** 1 Department of Geography, Western University, 1151 Richmond Street, Room 3213 SSC, London, ON N6A 5C2 Canada; 2 Whakauae Research for Maori Health and Development, 60 Ridgway Street, Whanganui, 4541 New Zealand; 3 Te Kupenga Hauora Māori, University of Auckland, Auckland, 1142 New Zealand

**Keywords:** Public health, Climate change, Indigenous, Competencies, Feminist, Santé publique, changement climatique, Autochtones, compétences, féminisme

## Abstract

Indigenous peoples have long been successful at adapting to climatic and environmental changes. However, anthropogenic climatic crisis represents an epoch of intensified colonialism which poses particular challenges to Indigenous peoples throughout the world, including those in wealthier ‘modern’ nation states. Indigenous peoples also possess worldviews and traditional knowledge systems that are critical to climate mitigation and adaptation, yet, paradoxically, these are devalued and marginalized and have yet to be recognized as essential foundations of public health. In this article, we provide an overview of how public health policy and discourse fails Indigenous peoples living in the colonial nation states of Canada and Aotearoa New Zealand. We argue that addressing these systemic failures requires the incorporation of Indigenous knowledges and Indigenous feminist perspectives beyond superficial understandings in public health-related climate change policy and practice, and that systems transformation of this nature will in turn require a radical revision of settler understandings of the determinants of health. Further, public health climate change responses that centre Indigenous knowledges and Indigenous feminist perspectives as presented by Indigenous peoples themselves must underpin from local to global levels.

## Résumé

Les peuples autochtones ont de tout temps réussi à s’adapter aux changements du climat et de leur environnement. La crise climatique anthropogène constitue toutefois une époque de colonialisme intensifié qui pose des difficultés particulières aux peuples autochtones du monde entier, y compris ceux des États-nations riches et « modernes ». Les peuples autochtones possèdent aussi des visions du monde et des systèmes de savoir traditionnels indispensables aux efforts d’atténuation et d’adaptation au changement climatique; paradoxalement, ces visions et systèmes sont dévalués et marginalisés et ne sont pas encore reconnus comme étant des bases essentielles de la santé publique. Dans cet article, nous expliquons en général en quoi les politiques et le discours de la santé publique laissent sur le carreau les peuples autochtones vivant dans les États-nations coloniaux du Canada et d’Aotearoa (la Nouvelle-Zélande). Nous faisons valoir que pour aborder ces échecs systémiques, il faut intégrer les savoirs autochtones et les perspectives féministes autochtones au-delà d’une compréhension superficielle des politiques et des pratiques de santé publique relatives au changement climatique, et qu’une telle transformation des systèmes exigera en retour une révision radicale des savoirs coloniaux sur les déterminants de la santé. Plus encore, les ripostes de la santé publique au changement climatique, que ce soit à l’échelle locale ou mondiale, doivent être centrées sur les savoirs autochtones et les perspectives féministes autochtones tels que présentés par les peuples autochtones eux-mêmes.

## Introduction

As industrialized colonial nation states facing anthropogenic climate change, Canada and Aotearoa New Zealand (ANZ) share several characteristics in common. Each country has an extraordinarily high carbon footprint per capita—Canada largely due to intensive fossil fuel production and consumption and ANZ more due to its intensive agriculturally dependent economy—amid rapidly changing human-ecological conditions. Both nation states also have significant Indigenous populations who are grappling with the realities of ongoing colonization, including glaring health, wealth and power disparities relative to their non-Indigenous populations, and who, for these and other reasons, are particularly vulnerable to the impacts of climate crisis. Reflecting on our knowledge as Indigenous scholars from these two nation states, a central tenet of our paper is that anthropogenic climate change is intimately connected to the ideologies, systems and practices of colonialism and that the impacts on Indigenous peoples represent an intensification of colonialism (Whyte 2017; Jones 2019). Underlying dynamics include intensified forms of patriarchy, western scientific imperialism, aggressive neo-liberalism and subsequent environmental dispossession, and ongoing acculturation (Jones et al. 2014; Williams et al. 2018; Jones 2019). Climate change is no longer ‘the problem’, rather it is a key risk amplifier to the accelerating decline of the earth’s ecosystems (UNDRR 2019). While reducing carbon emissions and other technologically driven approaches to the climate crisis are vital to sustaining planetary life, more fundamental changes are required (Bendell 2018).

The response to these issues has often been driven by similar sets of dynamics and actors as those responsible for the problems in the first place. While it is generally accepted that Indigenous knowledges (IK) hold much significance for climate change strategies, culturally dominant Eurocentric social structures, norms and conventions ensure their marginalization at broader and more influential decision-making levels (Williams et al. 2018). This is equally the case in public health policy. In general, Indigenous peoples continue to be viewed through a deficit lens on the basis of their vulnerability. Because of this, Indigenous worldviews and cultural practices are seen as being relevant solely to improving their own health and well-being, rather than as a vital knowledge-base for a shared planetary future. Indeed, the Mi’kmaq have a sovereign law, once unspoken, called ‘*netukulimk*’, a concept that has provided moral and spiritual guidance about the sustainable use of the earth’s resources, provided as gifts from the Creator, in a way that ensures future generations can be provided for (Prosper et al. 2011).

In April 2019, hundreds of Indigenous delegates, including the authors, along with their non-Indigenous colleagues, gathered at the 23rd International Union for Health Promotion and Education (IUHPE) World Conference on Health Promotion in ANZ. Here, the Indigenous delegates responded to the conference theme “Waiora: Promoting Planetary Development and Sustainable Health for All” with a call to action for “global health promotion…to make space for and privilege Indigenous peoples’ worldviews and IK in promoting planetary health…” (International Union for Health Promotion and Education 2019). Our response to this call is to interrogate what this call to action means for the public health community that is invested in climate change, but within which Indigenous worldviews and knowledges remain largely excluded.

Our commentary advocates for a radical revision of the global public health response to climate change. Pivotal to this is recognition by the public health community that Indigenous worldviews and knowledges are key to collectively navigating our way through the turmoil of anthropogenic climate crisis. Focusing primarily on the Canadian and ANZ public health contexts, we begin to lay the groundwork for a public health response to climate change. This is grounded in the primacy of Indigenous, land-based epistemologies and knowledges becoming key drivers in a public health-related discourse. Such discourses must be led by Indigenous peoples who possess the necessary forms of expertise to drive such fundamental change.

### Public health policy must decolonize

The Public Health Agency of Canada (PHAC) recognizes social determinants of health (SDoH) as influencing the health of populations (i.e., income, education, gender, culture) (Government of Canada 2019). This framework has been critiqued by Indigenous scholars for its failure to consider the unique structural determinants of health that colonization, assimilative policies, and Indigenous-settler relations have created (Reading 2018). PHAC further recognizes ecological determinants of health (EDoH), which are intended to highlight the interdependence of environment and health. However, PHAC’s framing of the EDoH as the ‘goods and services’ that humans get from nature (Canadian Public Health Association 2015, p. iv) is antithetical to relational Indigenous epistemological orientations. We posit IK as the ‘exemplar’ for EDoH, beyond the social, structural and ecological determinants of PHAC, because IK is grounded in the interconnectedness of humans with nature (Buse et al. 2018).

In ANZ, Mātauranga Māori (Māori Knowledge) realities are similarly positioned at the margins of public health policy. Mātauranga Māori has enabled Indigenous peoples to live in balance with their social and natural environment over many centuries. One reason for Mātauranga Māori’s marginalization is that it is mistakenly viewed as ‘static’, solely focused on traditional aspects of knowledge grounded in the past, rather than a dynamic and developing body of knowledge with much relevance to contemporary public health policy and discourse (Prussing and Newbury 2016). Came et al. (2019) note that while there appears to be a high commitment to engagement with Mātauranga Māori in health policy, the knowledge is undervalued and the application is often tokenistic.

In both countries, these systemic biases play out at local levels in terms of everyday public health practice. For example, the *Core Competencies for Public Health in Canada* (developed by PHAC) which include “knowledge, skills and attitudes necessary for effective public health practice in all health disciplines” make no mention of colonialism as a factor to consider in public health practice (Hunt and National Collaborating Centre for Aboriginal Health 2015, p. 5). Indeed, public health policy continues to lack a focus on Indigenous health and well-being despite known disparities in social and structural determinants of health (Richmond and Cook 2016). Similarly, in ANZ, despite the formulation of *Generic Competencies for Public Health* (2007) which incorporate recognition of Māori as tangata whenua (people of the land) who should self-determine our own needs, there is a monocultural approach to health policy which continues to marginalize Māori worldviews and knowledge. Western values become the default in the determination of competency (Came 2014).

Further, changes wrought by climate change are generally exacerbating the colonial-rooted inequalities that Indigenous women have historically experienced, yet little research has been conducted from an Indigenous feminist perspective (Williams et al. 2018). In part in response to this, the Intergovernmental Panel on Climate Change (IPCC) has recognized the need for gender-sensitive adaptation and mitigation strategies (IPCC 2018). In Canada, Williams et al. (2018) note that the gendered aspects of climate change are remarkably absent in climate strategies. For instance, the Pan-Canadian Framework on Clean Growth and Climate Change Second Annual Synthesis Report on the Status of Implementation makes no mention of gender (Government of Canada 2018). Despite providing guidance on gender-based analysis of federal initiatives, the Federal government itself has failed to action this for climate change adaptation and mitigation policies. A scan of the ANZ public health-related literature relating to climate change by one of the authors suggests gender-based analysis has remained remarkably absent (Office of the Minister for Climate Change 2009), with recent advocacy for inclusion of gender equality (NCWNZ 2019) in the country’s Zero Carbon Amendment Bill.

Indigenous women in both countries are very active in grass roots climate change action and hold considerable ecological knowledge and insights that could inform related policies, yet, their leadership is often not visible in formal institutional levels of governance. Moreover, the critiques offered by Indigenous women scholars are predominantly represented in the so-called soft sciences and so are often subsumed at the bottom of the knowledge hierarchy in what Williams et al. (2018) refer to as the “masculinist” world of Western climate change science (Williams et al. 2018; p. 269). Further, we argue that framing climate change (and ecological destruction more broadly) as being rooted in the colonial capitalist *patriarchy* allows for understanding and addressing gendered violence against our Mother Earth.

## Conclusion

It is generally well understood that Western science and institutions continue to grapple with the necessity of drawing on IK, or acknowledging the interconnectedness between the health of humans, the land, and the environment (Ratima et al. 2019). Within public health, we are similarly being challenged to decolonize; this necessarily must begin with a recognition that our concepts of social and ecological determinants of health framework are grounded in the values of western society (Pyett et al. 2008) and not Indigenous values. At the minimum, we ask for the PHAC to recognize and disinvest in the futurity of a system that perpetuates a “modern-colonial habit of being” that has created, and continues to create, harms and instead make space for new possibilities (Stein 2019, p. 200). For instance, the Core Competencies for Indigenous Public Health Evaluation and Research (CIPHER) Program, developed by Indigenous scholars from the CANZUS^1^ nations to ensure the integration of Indigenous perspectives into public health education, practice, and governance (Baba and Reading 2012, p. 125), now guides the Master of Public Health Indigenous Health Program at the Waakebiness-Bryce Institute for Indigenous Health in the Dalla Lana School of Public Health (DLSPH) at the University of Toronto. This program trains graduates to allow Indigenous peoples to take the lead in the development of health policy and programming from an Indigenous lens with an emphasis on Indigenous-led/created theories (DLSPH and Mashford-Pringle 2019). One way forward may be to look to the CIPHER Program, which can be adapted to guide public health climate change policy and practice.

Institutions of higher education rarely import IK into western frames of reference; when done, it often results in its misrepresentation and misappropriation (Stein 2019). As stated in the IUHPE Waiora Statement’s calls to action, Indigenous people can and will speak for themselves—therefore, what is required of mainstream public health is the ability to listen. A genuine understanding and acknowledgement of IK and Indigenous women’s knowledges specifically will require nothing less than a re-imagination of the entire basis for our civilization over the past 400 years. Any refusal to undertake a radical revision of the public health response to environmental crisis that centralizes Indigenous worldviews and knowledges is to perpetuate colonization, leading us further down the pathway towards global ecological collapse.

## Footnotes

1 CANZUS—Canada, Australia, New Zealand, and the United States (Anglo-settler states).
